# Supplementary material for: Complex‐centric proteome profiling by SEC‐SWATH‐MS
Source: Mol Syst Biol. 2019 Jan 14;15(1):e8438. doi: 10.15252/msb.20188438 (PMC6346213; doi:10.15252/msb.20188438)
Supplement: Supplementary file 8 — Dataset EV7 [file MSB-15-e8438-s008.zip › feature_plots_string/Q9UJX6.pdf]

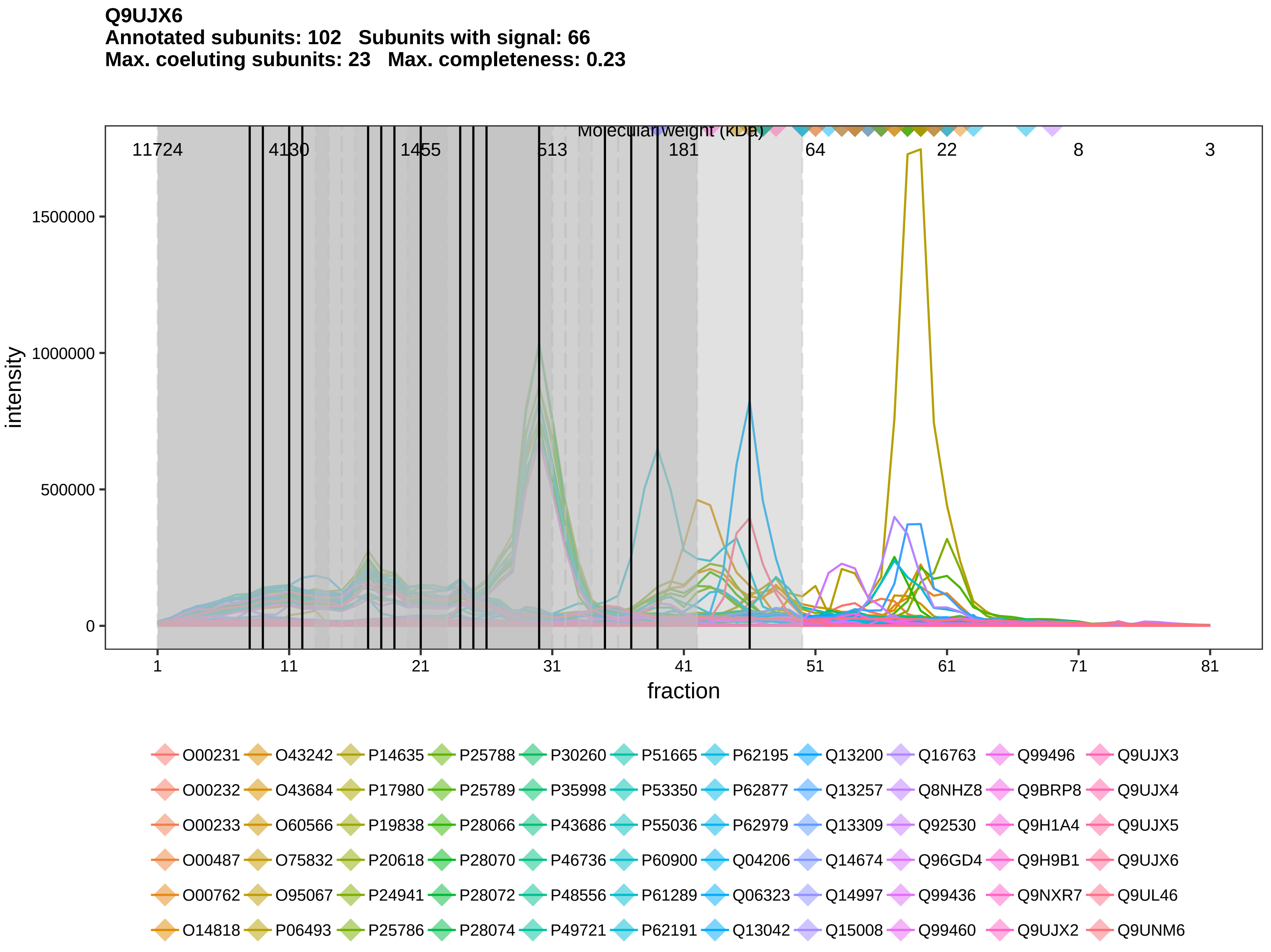

Q9UJX6
Annotated subunits: 102 Subunits with signal: 66
Max. coeluting subunits: 23 Max. completeness: 0.23
Molecular weight (kDa)
11724
4130
1455
513
181
64
22
8
3
intensity
1500000
1000000
500000
0
fraction
1
11
21
31
41
51
61
71
81
O00231 O43242 P14635 P25788 P30260 P51665 P62195 Q13200 Q16763 Q99496 Q9UJX3
O00232 O43684 P17980 P25789 P35998 P53350 P62877 Q13257 Q8NHZ8 Q9BRP8 Q9UJX4
O00233 O60566 P19838 P28066 P43686 P55036 P62979 Q13309 Q92530 Q9H1A4 Q9UJX5
O00487 O75832 P20618 P28070 P46736 P60900 Q04206 Q14674 Q96GD4 Q9H9B1 Q9UJX6
O00762 O95067 P24941 P28072 P48556 P61289 Q06323 Q14997 Q99436 Q9NXR7 Q9UL46
O14818 P06493 P25786 P28074 P49721 P62191 Q13042 Q15008 Q99460 Q9UJX2 Q9UNM6
